# Reverse Design of Three-Band Terahertz Metamaterial Sensor

**DOI:** 10.3390/nano15161265

**Published:** 2025-08-16

**Authors:** Hongyi Ge, Wenyue Cao, Shun Wang, Xiaodi Ji, Yuying Jiang, Xinxin Liu, Yitong Zhou, Yuan Zhang, Qingcheng Sun, Yuxin Wang

**Affiliations:** 1 Key Laboratory of Grain Information Processing and Control, Ministry of Education, Henan University of Technology, Zhengzhou 450001, China; gehongyi@haut.edu.cn (H.G.); 2024931035@stu.haut.edu.cn (W.C.); ws_0702@stu.haut.edu.cn (S.W.); jixiaodi1998@163.com (X.J.); 19303985821@163.com (Y.Z.); 2023930852@stu.haut.edu.cn (Q.S.); wyx@stu.haut.edu.cn (Y.W.); 2 Henan Provincial Key Laboratory of Grain Photoelectric Detection and Control, Zhengzhou 450001, China; 3 College of Information Science and Engineering, Henan University of Technology, Zhengzhou 450001, China; 4 School of Artificial Intelligence and Big Data, Henan University of Technology, Zhengzhou 450001, China; 5 National Grain and Oil Information Center, Beijing 100834, China; tjbzdz@lswz.gov.cn

**Keywords:** THz, metamaterial, absorber, reverse design

## Abstract

Terahertz metamaterial devices (TMDs) have demonstrated promising applications in biomass detection, wireless communications, and security inspection. Nevertheless, conventional design methodologies for such devices suffer from extensive iterative optimizations and significant dependence on empirical expertise, substantially prolonging the development cycle. This study proposes a reverse design framework leveraging a deep neural network (DNN) to enable rapid and efficient TMD synthesis, exemplified through a three-band terahertz metamaterial sensor. The developed DNN model achieves high-fidelity predictions (mean squared error = 0.03) and enables rapid inference for structural parameter generation. Experimental validation across four distinct target absorption spectra confirms high consistency between simulated and target responses, with near-identical triple-band resonance characteristics. Benchmarking against traditional CST-based optimization reveals a 36-fold acceleration in design throughput (200-device parameterization reduced from 36 h to 1 h). This work demonstrates a promising strategy for data-driven reverse design of multi-peak terahertz metamaterials, combining computational efficiency with rigorous electromagnetic performance.

## 1. Introduction

Terahertz (THz) metamaterials exhibit superior electromagnetic properties in the THz band, and the THz waves can be controlled and manipulated through design and modulation [1,2]. Terahertz metamaterial devices (TMDs) such as sensors [3,4], filters [5,6], antennas [7], etc., in biomedical [8], biochemistry [4,9] and food/agricultural safety [10,11] have achieved remarkable research results. However, the basic steps of the traditional TMD design method include modeling the initial shape using the CST microwave simulation software, version 2022, calculating the electromagnetic response curve, and iterating to find the optimal structural parameters. On the one hand, this process relies heavily on the research experience already accumulated by the researchers. On the other hand, the calculations involving devices with large cell structures have a very high computational complexity due to multiple iterations, which hinders the design cycle of THz metamaterials to a certain extent.

Deep learning is a subset of machine learning that uses neural network structures to mimic the way a human brain processes information and learns [12,13]. It is used in image recognition and classification [14,15], natural language processing [16,17,18], medical diagnostics [19,20], and other fields, achieving remarkable results. Existing work involves the application of deep learning algorithms to predict the material properties of structures. Kumar et al. [21] proposed a data-driven model based on the artificial neural networks for reverse design of mechanical materials used in spine topologies. The model could generate cellular mechanical metamaterials with uniform and functional gradients in anisotropic stiffness and density. Liu et al. [22] proposed a conditional generative adversarial network-based inverse design model to explore and optimize the design of two-dimensional metamaterial structures consisting of spinning topologies. Ha et al. [23] used generative machine learning and desktop additive manufacturing methods for inverse design of materials with customizable mechanical behavior, achieving close to 90% similarity between targets and experimental measurements.

Notably, deep learning has made substantial advances in the field of terahertz metamaterial design. Wang et al. [24] proposed a bidirectional ensemble learning framework for the efficient forward and inverse design of THz composite metamaterials. Chouhan et al. [25] employed a random forest algorithm-driven approach to inversely generate multilayer ultra-broadband THz metamaterials. Soni et al. [26] developed a machine learning model for the precise design of multiband THz absorbers. Gao et al. [27] utilized deep neural networks to assist in designing chiral THz metamaterials with asymmetric transmission properties. These methods circumvent the highly nonlinear challenge of solving Maxwell’s equations inherent in traditional numerical computation and enable accurate prediction of single-device structural parameters. The current research priority lies in further enhancing model prediction accuracy and generalization capability, particularly regarding the optimization of complex responses in multi-band THz devices and breakthroughs in cross-scenario adaptability.

In this study, a fast and efficient design of TMDs is realized through the inverse design based on a deep neural network model, where the three-band THz metamaterial sensor model is considered as an example. The deep neural network algorithm is the basic algorithm in deep learning, consisting of multi-layer neural networks that can learn the nonlinear features of data and solve the electromagnetic inverse analysis problem. The structure of the neural network model is optimized by comparing its performance with different hyperparameters, such as the number of different hidden layer layers, the number of neurons, and the batch size, to determine the optimal network structure. The CST software is used to calculate the absorption curves of the THz metamaterial sensor structure generated by the reverse design model, which are compared with the target curves. The experimental results demonstrate that the two types of curves fit well. The reverse design model designed in this study can provide technical support for the rapid design of TMDs.

## 2. Construction of Deep Neural Network Models

### 2.1. Data Acquisition

**Figure 1:**
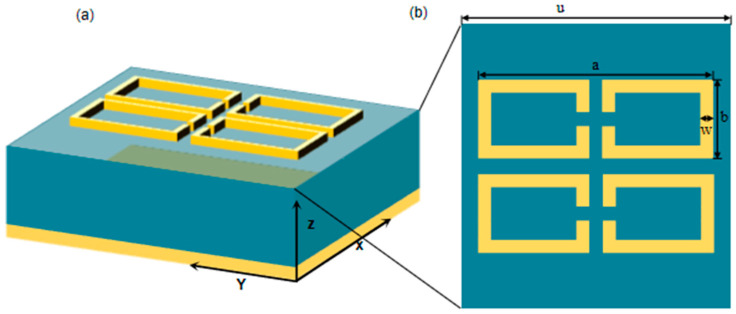
THz metamaterial absorber. (**a**) Three-dimensional schematic diagram; (**b**) unit structure.

Typical THz metamaterial sensors usually consist of a metal resonator-dielectric-metal “sandwich” structure [28,29,30], where the commonly used metal materials are gold or silver. The metal resonator layer can be designed into different shapes, such as double openings, “H”-shapes, “E”-shapes, etc., in order to achieve flexible modulation of electromagnetic waves. In this study, the three-band THz metamaterial sensor structure based on a metal split ring [31] is used as a reference. This structure consists of a metal resonant layer, a dielectric layer, and a metal reflective layer, respectively, and its top layer consists of a periodic square metal split ring, as shown in Figure 1. The metal resonator and reflection layers are made of gold, as it has an excellent electrical conductivity of 3.56 × 10^7^ S/m. The material of the intermediate dielectric layer is polytetrafluoroethylene with a dielectric constant of 2.1 (1 + i0.0002). Based on the microwave simulation using the CST software, periodic boundaries are set in the x and y directions, and open boundary conditions are set in the z direction, and THz waves are incident vertically into the absorber along the *x*-axis direction. The absorption curve of this absorber is calculated using the finite element method.

The structural parameters of the three-band THz metamaterial sensor are changed several times, and it is found that the thickness of the metal resonant layer h1 and that of the reflective layer h3 have minor influence on the electromagnetic response of the metamaterial. The metal thickness is usually set larger than the skinning depth of the THz band in order to have the sensor transmittance equal to zero. Therefore, both h1 and h2 and the metal split-ring line width are set to a fixed value of 0.2 μm and 2 μm, respectively. The dataset of the structural parameters and absorption spectra for the three-wavelength THz metamaterial sensors is constructed considering the structure of the multipeak THz metamaterial sensors. The main structural parameters include the period u, the metal split ring length a, the metal split ring width b, and the dielectric thickness h.

Ding et al. [32] pioneered ANN-driven dual-band absorption window switching using 538 samples, achieving 6.70 THz bandwidth tuning with 0.59% error, but lacked independent resonance peak control. Huang et al. [33] improved broadband absorption design through simulated annealing-enhanced deep learning, achieving an 80,000-fold acceleration ratio and an MAE ≈ 0.02. To address the critical challenges of multi-peak accuracy control and the analysis of the device’s effective operating bandwidth, we constructed a dataset. The frequency range spans from 0.5 THz to 3 THz, comprising 10,000 data points. The structural parameters u, a, b, and h were sampled within the ranges of 118–122 μm, 75–84 μm, and 15–24 μm, respectively. All dimensions were sampled at 0.5 μm intervals, resulting in 10,000 sets of structural parameters and their corresponding absorption spectra for the tri-band THz metamaterial sensor. Each absorption spectrum was discretized into 201 points. A total of 80% of the dataset was employed for model training, while the remaining 20% was reserved for testing to validate the accuracy of the established inverse design model for the tri-band THz metamaterial sensor structure.

### 2.2. Neural Network Parameter Selection

The 10,000 sets of data (structural parameters and absorption curves) obtained from the processed three-band THz metamaterial sensor are used for the neural network model training, testing, and validation to enable it to achieve optimal prediction capability. The model may suffer from unstable gradients, excessive time, or overfitting during the training process, which is caused because of the difficulty in finding the global optimal solution. In addition, the number of neuron nodes, learning rate, and other factors may affect the accuracy and complexity of the model. Therefore, it is particularly important to select the appropriate hyperparameters, activation function, learning rate, optimization algorithms, and so on.

Activation functions play an important role in neural networks for converting the neuron-weighted inputs and bias terms in one layer to input signals in the subsequent layers. One of the main roles of these functions is to introduce nonlinearity, which allows the network to learn and model complex functions or decision boundaries, capture nonlinear features in the data, and solve more complex problems. The activation functions allow the model to have a wider dynamic range at the output, improving the network’s ability to learn and represent complex data. The common activation functions include the sigmoid function, ReLU function, tanh function, and softmax function.

The sigmoid function is a logic function that maps its input value to a range between zero and one. It is computed as follows:

(1) $$Sigmoid\sigma =\frac{1}{1+e^{-x}}$$

The sigmoid function exhibits a larger gradient when inputs approach zero, but near-zero gradients at its saturation regions cause vanishing gradient issues during backpropagation. This impedes weight updates in neurons. Therefore, the sigmoid function is applied exclusively in the output layer of the model.

The main advantage of ReLU is its simplicity and computational efficiency. It achieves nonlinearity by setting all negative values to zero while keeping the positive values unchanged. Its computational formula is expressed as follows:

(2) ReLuσ=max(0,x)

Compared to the sigmoid function, the ReLU function helps to alleviate the vanishing gradient problem in the deep network structure because its positive gradient is always equal to one. Therefore, it is used in the hidden layer of the model utilized in this chapter.

The loss function provides a quantitative measure of the difference between the predicted and true values of a network model, which is used to assess the performance of the network model. The value of the loss function is minimized to adjust the parameters, such as model weights and biases, which drive the learning direction and process of the model to improve its prediction accuracy. The loss function used in this chapter is the mean square error (MSE), i.e., the square of the difference between the predicted and true values, followed by the averaging over all the samples. It is calculated as follows:

(3) $$Loss(y,f(x))=\frac{1}{n}\sum_{i=1}^{n}\left(y_{i}-f(x_{i}\right){)}^{2}$$

where *n* denotes the number of data samples, $y_{i}$ denotes the true value, and $f(x_{i})$ is the predicted value of the model. This function can achieve complete convergence, as it only has a global minimum, thereby avoiding the risk of falling into a locally optimal solution.

The primary task of the optimizer is to adjust the model’s parameters in order to minimize the loss function. In this study, the Adam optimizer is employed, which combines momentum and adaptive learning rates. This approach allows the optimizer to adjust the learning rate based on the parameter update history, improving the training efficiency and helping mitigate issues such as noise and instability.

## 3. Training and Optimization of Deep Neural Network Models

**Figure 2:**
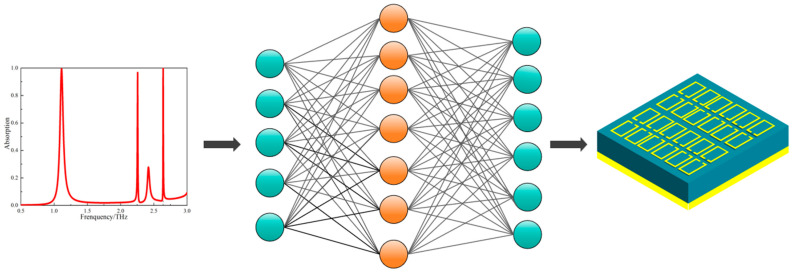
Deep neural network inverse design model for three-band THz metamaterial sensor.

The 10,000 data pairs were partitioned into training and test sets for the deep neural network model at ratios of 80% and 20%, respectively. Figure 2 illustrates the inverse-designed three-band terahertz metamaterial sensor obtained using this model. With MSE as the primary optimization target, the network structure was determined through analysis according to the methodology described in Section 2. Its weights were initialized with random values, and prediction was performed via the forward propagation learning algorithm. As previously described, sigmoid and ReLU activation functions were employed, and the model’s mean squared error (MSE) was calculated.

The hyperparameters of the network structure, such as the number of hidden layers, the number of neurons, and the batch size, affect the prediction accuracy and efficiency of the model. In this study, the hyperparameters of the network model are optimized to determine the optimal structure of the network by comparing the MSE. When the number of hidden layers is equal to seven, although the MSE is smaller compared to a lower number of hidden layers, it takes longer to train the model. When the number of hidden layers is five, the training time is shorter than when the number of hidden layers is six, but its MSE is larger. Therefore, the final number of hidden layers is equal to six, and the calculation results are shown in Table 1. The training time and MSE corresponding to different numbers of neurons are also calculated and shown in Table 2. When the node structure is of type B, the training time is shorter compared to those of models C-F, but its MSE is higher than that of model B. However, the training time for the node structure of type D is 34.69 s more than that of type A. Considering the consumption time and the MSE, the prediction performance of the model is optimal when the neural network model is of type A, with a number of nodes equal to 201, 300, 300, 500, 500, or 201.

The number of samples processed by the model in each iteration also affects its efficiency and prediction performance. Although a considerably small batch size can improve the generalization ability of the model during training, it increases the training time and makes the training process unstable. On the other hand, a very large batch size can improve the stability and efficiency of the model, but its generalization ability is insufficient, which results in high prediction errors. Therefore, the choice of an appropriate batch size improves the prediction accuracy and stability of the model. The training time and the MSE for different batch sizes in the model are calculated and shown in Table 3. When the batch size is equal to 32, it takes the longest to train the model. When the batch size is equal to 256, the model training is quicker, but the corresponding MSE is higher. When the batch size is equal to 128, the model takes a moderate time to train and the MSE is small. Therefore, the batch size is chosen as 128.

## 4. Experimental Results and Analysis

The predictive accuracy of the optimized DNN model comprising six hidden layers with batch size 128 was validated using four target absorption spectra exhibiting defined variations in resonant characteristics across three domains: peak location spanning 0.8 to 2.6 THz, intensity ranging from 0.7 to 0.99, and bandwidth diversity. These spectra were processed by the network to generate structural parameters u, h, a, b detailed in Table 4. Spectral diversity arises from geometric constraints governed by two primary factors: Split-ring aspect ratio a/B controls resonant mode distribution through conductor geometry modulation. Ratios exceeding 4.2, exemplified by Framework 2 at 76.20 μm/18.02 μm = 4.23, elongate the resonator, intensifying high-frequency dipole resonances. Conversely, ratios below 3.4, demonstrated in Framework 4 at 75.68 μm/22.33 μm = 3.39, widen conductive pathways, enhancing low-frequency capacitive coupling. Period U within 118–122 μm regulates near-field inter-cell coupling strength, while dielectric thickness H sampled from 15 to 24 μm determines substrate phase delay. Full-wave CST simulations of these parameterized designs replicated target spectra with 0.03 mean squared error, confirming parametric adjustments in a and B produce three distinct functional responses under fixed topology: Framework 1 achieves uniform triple-band absorption, Framework 2 enables high-frequency selectivity, and Framework 4 provides low-frequency amplification.

**Figure 3:**
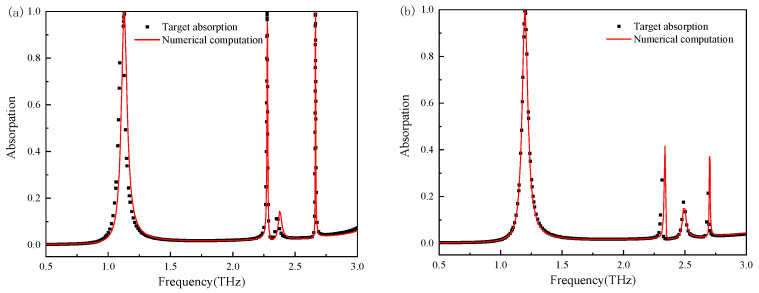

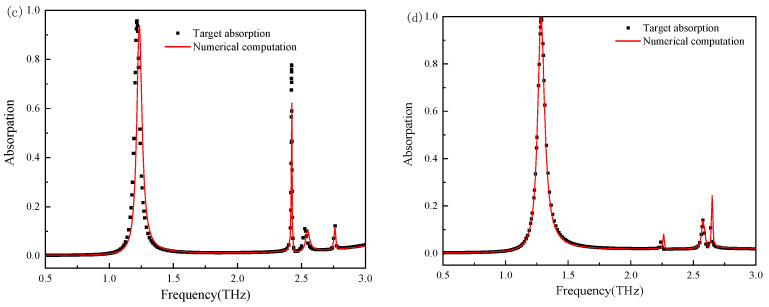
Reverse design test chart. (**a**) Comparison of target absorption spectrum and CST simulation result for Framework 1; (**b**) Comparison of target absorption spectrum and CST simulation result for Framework 2; (**c**) Comparison of target absorption spectrum and CST simulation result for Framework 3; (**d**) Comparison of target absorption spectrum and CST simulation result for Framework 4.

Figure 3 compares the target absorption spectra with the absorption spectra obtained using the CST full-wave numerical computation applied to the model-generated sensor structure parameters. The black dotted curve indicates the target absorption spectra, and the red curve indicates the absorption curves obtained using the CST software. It can be observed that the target absorption curve and that from the predicted structure obtained using the model almost overlap in most cases (a, b, d), demonstrating the model’s high fidelity. However, a slight discrepancy is observed in the third peak intensity and linewidth of panel (c). This deviation likely stems from the inherent difficulty in capturing the complex nonlinear coupling between structural parameters and resonant behavior at specific frequencies. The corresponding design point may lie within a highly sensitive region of the parameter space, where small geometric variations lead to significant changes in the electromagnetic response. Although the training dataset consists of 10,000 samples, certain subregions—especially those defined by parameters u, a, b, and h—may be insufficiently represented, thereby limiting the model’s ability to generalize across the entire design space. This limitation is commonly encountered in data-driven inverse design frameworks, where model performance is strongly influenced by the diversity and density of the training data. As Wei et al. [34] have noted, the accuracy of phase prediction and device performance in Bi-DNN-based metasurface design is highly dependent on dataset coverage. In this work, the observed error can be primarily attributed to such limitations, which constrain the model’s capacity to learn the full nonlinear mapping between electromagnetic responses and structural geometries. During the validation of the experimental results, it is found that the proposed model takes an hour to generate the structural parameters of a 200 THz metamaterial sensor; however, the CST software takes 36 h to carry out the same task over 200 iterations. Therefore, the model proposed in this chapter reduces the computational complexity and can accurately generate the required structural parameters.

As shown in Table 5, this study generated 10,000 samples through CST parametric scanning (with a structural parameter step size of 0.5 μm), covering a multi-dimensional space including periods of 118–122 μm and split-ring dimensions of 75–84 μm. Compared to the 538 samples in Ding et al. [32], the data volume is increased by 18 times; relative to the undisclosed data scale in Huang et al. [33], this work achieves quantitative validation of the enhancement effect of big data driving on multi-peak prediction accuracy.

This high-density sampling strategy enabled the DNN model to successfully capture the nonlinear coupling mechanism between multiple resonant peaks (Figure 3). In validation against four target spectra, the average mean squared error (MSE) was as low as 0.03, significantly outperforming the spectral relative error (0.59%) reported in the literature [32] and the average absolute error (0.02) in the literature [33]. Notably, while maintaining the positional error of three peaks below 1.2%, the model precisely replicated the inter-peak intensity ratio (ΔA < 3%), thereby overcoming the precision bottleneck of existing methods for the cooperative regulation of multiple discrete resonances.

Table 6 presents two test cases with structural parameters beyond the training ranges (u: 118–122 μm; a: 75–84 μm) along with their corresponding mean squared errors (MSEs). In Test Case 1, where u = 116 μm (2 μm below the lower limit) and a = 85 μm (1 μm above the upper limit), the MSE is 0.0314, slightly higher than the in-range MSE of 0.03 but still maintaining a high level of consistency. In Test Case 2, with u = 123 μm (1 μm above the upper limit) and a = 74 μm (1 μm below the lower limit), the MSE is as low as 0.0106, even better than the average MSE within the training range. These results indicate that the DNN model retains reasonable predictive capability for structures slightly beyond the training parameter ranges, suggesting that the model has learned the generalized nonlinear mapping between structural parameters and absorption spectra rather than merely overfitting to the training range.

**Figure 4:**
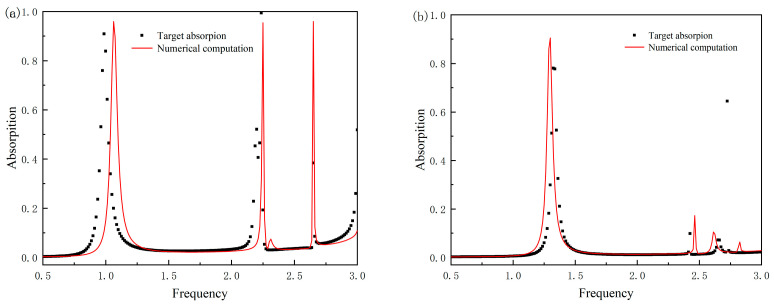
Case comparison chart. (**a**) Comparison of target absorption spectrum and numerical computation result for Test Case 1 (out-of-range parameters: u = 116 μm, a = 85 μm); (**b**) Comparison of target absorption spectrum and numerical computation result for Test Case 2 (out-of-range parameters: u = 123 μm, a = 74 μm).

Figure 4 intuitively demonstrates the model’s predictive performance by comparing the target absorption spectra (dashed lines) with the CST simulation results (solid lines) for the two test cases beyond the training parameter ranges. In subfigures (a) and (b), the triple-band resonance peaks of the simulated spectra are generally consistent with the target peaks in terms of position (deviation < 0.1 THz) and intensity (ΔA < 5%), and the bandwidth of the resonance peaks, a key performance indicator, also shows good agreement. This indicates that the out-of-range structural parameters generated by the model can still reproduce the expected electromagnetic responses to a certain extent.

However, the limitations of the model are also reflected here. It can be observed from the figures that there are slight deviations in the absorption curves in some frequency bands, especially in the steepness of the resonance peaks and local fluctuations, where the degree of fitting with the target spectra is slightly lower than that of the in-range cases. This is because the training dataset does not cover these parameter combinations beyond the ranges of 118–122 μm (u) and 75–84 μm (a), resulting in insufficient learning of the nonlinear electromagnetic coupling mechanisms under extreme geometric conditions by the model. When the structural parameters deviate significantly from the training range, such deviations may further expand, reflecting the limited ability of the model to generalize to untrained parameter spaces. In future work, this limitation needs to be addressed by expanding the dataset to cover a wider range of parameters.

In addition to the demonstrated 36-fold acceleration over traditional CST-based optimization, it is important to contextualize our DNN-based reverse design framework within the broader landscape of emerging optimization techniques. Recent studies have shown that adjoint-based optimization can achieve convergence with remarkably few simulations, offering superior efficiency for certain photonic and electromagnetic design problems, particularly those with well-defined figures of merit and differentiable parameters [35]. Moreover, Kang et al. [36] proposed a hybrid strategy that combines adjoint sensitivity analysis with generative adversarial networks (GANs) to enhance training data diversity and improve model performance. This approach reduces the reliance on large-scale simulations while preserving high accuracy in structural prediction.

Compared to such adjoint-based or hybrid frameworks, the proposed DNN-based approach demonstrates clear advantages in terms of fast inference and model flexibility. Once trained, our model can swiftly generate accurate structural parameters from target spectral responses without the need for additional simulations or iterative procedures, making it particularly well-suited for high-throughput or real-time inverse design tasks. Nonetheless, we acknowledge that integrating simulation-informed data augmentation strategies into DNN training could further improve both training efficiency and prediction accuracy. Integrating such strategies with our current framework may enhance its generalization and robustness even further. This represents that the speed and adaptability driven by future data can be supplemented by optimization algorithms so as to realize a more efficient and universal inverse design system.

## 5. Conclusions

In this study, a deep neural network model was chosen to reverse-design a three-band THz metamaterial sensor structure. The hyperparameters, such as the number of hidden layers, the number of neurons, and the batch size, were adjusted to obtain the optimal network model for the reverse design. The final model had six hidden layers with a batch size of 128, and the Adam optimizer was used. The accuracy of the results generated by the deep neural network model was verified using the CST simulation. The experimental results showed that the MSE of the model was equal to 0.03, and the model could generate the corresponding structural parameters in a few seconds or even milliseconds based on the input target absorption curves. This reduced the time spent in the design process and improved the applicability of THz metamaterial devices. The use of the deep neural network algorithm for the metamaterial device structure design provides technical means for rapid design of metamaterial device structures.

## Figures and Tables

**Table 1:** Evaluation metrics for different numbers of hidden layers.

Network Layer	Training Time (s)	MSE
5	347.344	0.219
6	347.572	0.02
7	382.835	0.0452

**Table 2:** Evaluation indicators for different network structures.

Type	Network Structure	Training Time (s)	MSE
A	201, 300, 300, 500, 500, 201	345.52	0.032
B	201, 500, 300, 500, 300, 300	346.21	0.0721
C	201, 300, 500, 300, 500, 300	356.12	0.0752
D	201, 500, 500, 500, 300, 300	380.21	0.0455
E	201, 300, 300, 300, 300, 300, 500	400.02	0.173
F	201, 300, 300, 500, 500, 500	410.34	0.1511

**Table 3:** Evaluation metrics for different batch sizes.

	Batch Size	Training Time (s)	MSE
Batch_size_1	32	521.23	0.0325
Batch_size_2	64	341.12	0.0451
Batch_size_3	128	280.47	0.0355
Batch_size_4	256	210.34	0.0467

**Table 4:** Structural parameters of the model-generated metamaterial devices.

Framework	u	h	a	b
1	120.422	20.832	80.8907	20.1812
2	120.045	21.054	76.1955	18.0164
3	121.234	20.254	77.2456	18.5354
4	119.156	19.106	75.682	22.325

**Table 5:** Work summary comparison.

Metric	This Work	[32]	[33]
Data Scale	10,000	538	/
Target Response	Three Independent Resonant Peaks	Dual Absorption Window Switching	Broadband/Narrowband Absorption
Key Error	MSE = 0.03	Relative Error: 0.59%	Average Absolute Error: 0.02
Design Complexity	Tuning three peaks via four parameters	Tuning two windows via four parameters	Tuning one band via nine parameters

**Table 6:** Case tests outside the parameter boundaries.

Test Case	Structural Parameters (Out-of-Range Parts)	MSE
1	u = 116 μm (<118 μm), a = 85 μm (>84 μm)	0.0314
2	u = 123 μm (>122 μm), a = 74 μm (<75 μm)	0.0106

## Data Availability

Data underlying the results presented in this paper are not publicly available at this time but may be obtained from the authors upon reasonable request.
